# Rationale, design, and methods for the Medical Optimization and Management of Pregnancies with Overt Type 2 Diabetes (MOMPOD) study

**DOI:** 10.1186/s12884-018-2108-3

**Published:** 2018-12-12

**Authors:** Diane C. Berry, Sonia Davis Thomas, Karen F. Dorman, Amber Rose Ivins, Maria de los Angeles Abreu, Laura Young, Kim Boggess

**Affiliations:** 1 The University of North Carolina at Chapel Hill School of Nursing, Campus Box 7460, Chapel Hill, NC 27599-7460 USA; 2 Department of Biostatistics, University of North Carolina, 137 E Rosemary St Suite 203, Chapel Hill, NC 27514 USA; 3 The University of North Carolina at Chapel Hill School of Medicine, Campus Box 7516, Chapel Hill, NC 27599-7516 USA

**Keywords:** Type 2 diabetes, Pregnancy, Maternal outcomes, Fetal outcomes

## Abstract

**Background:**

Annually in the US, over 100,000 pregnant women with overt type 2 diabetes give birth. Strict maternal glycemic control is the key to optimizing infant outcomes. Medical treatment of type 2 diabetes in pregnancy is generally restricted to insulin, as data on the safety and efficacy of oral hypoglycemic agents in pregnancy are limited. However, over one-third of infants born to women with type 2 diabetes experience an adverse outcome, such as premature delivery, large-for-gestational age, hypoglycemia, hyperbilirubinemia, or birth trauma, suggesting that current treatment regimens fall short of optimizing outcomes. Metformin is the pharmacologic treatment of choice for type 2 diabetes outside of pregnancy. Metformin is favored over insulin because it results in less weight gain, fewer hypoglycemic episodes, and is administered orally rather than injected. However, metformin is not typically used for treatment of type 2 diabetes complicating pregnancy, mainly because no large clinical studies have been conducted to examine its use in this context.

**Methods/design:**

This is a randomized double-blind multi-center clinical trial of insulin plus metformin versus insulin plus placebo for the treatment of type 2 diabetes complicating pregnancy. A total of 1200 women with type 2 diabetes will be randomized between 10 weeks 0 days’ and 20 weeks 6 days’ gestation and followed until 30 days after delivery. Neonate outcomes will be followed until 30 days of age. The primary aim is to compare the effect of insulin and metformin versus insulin and placebo on composite adverse neonatal outcomes, comprising perinatal mortality, preterm delivery, neonatal hypoglycemia, hyperbilirubinemia, large-for-gestational age small for gestational age, low birth weight, and/or birth trauma. Key secondary aims are to compare treatment groups for neonatal fat mass and rate of maternal hypoglycemia. Additional aims are to assess the side effects and safety of insulin and metformin among pregnant women with overt type 2 diabetes and to compare gestational weight gain among women treated with metformin plus insulin versus insulin alone.

**Discussion:**

Successful completion of this study will result in high-quality, contemporary evidence for management of overt type 2 diabetes complicating pregnancy to improve neonatal outcomes.

**Trial registration:**

NCT02932475 (05/17/2016).

## Background

Type 2 diabetes mellitus (T2DM) is the most common type of diabetes [1]. In the United States (U.S.), T2DM affects approximately 29.1 million people (9.3%), and, of those 29.1 million, 21.0 million are diagnosed and 8.1 million are undiagnosed [1]. Approximately two million women of reproductive age have T2DM [1]. Annually in the U.S. T2DM affects approximately 100,000 pregnancies [2]. By 2030, the number of women entering pregnancy with T2DM is expected to double [2]. T2DM is the most common type of overt diabetes complicating pregnancy and is a serious public health concern [1, 2].

As the physiologic changes in pregnancy are designed to supply glucose to the fetus and placenta, the pregnant state promotes maternal insulin resistance [3]. In the second half of gestation, the fetus grows several-fold in size, and the absolute of glucose utilization increases substantially [3]. Placental glucose transfer increases secondary to the increasing metabolic requirements of the fetus [3]. Also, placental hormones increase the transplacental glucose concentration gradient, increasing the maternal glucose concentration relative to the fetus [3]. Increasing insulin resistance and transient hypoglycemia between meals and at night due to continuous fetal draw sets the stage for transient postprandial hyperglycemia after meals [3]. Therefore, insulin resistance in T2DM significantly complicates pregnancy, and optimal maternal glucose control is challenging.

Type 2 diabetes is associated with significant maternal and infant morbidity [2]. Type 2 diabetes in pregnancy is associated with adverse pregnancy outcomes, including miscarriage, congenital birth defects, fetal growth disturbance (under and over), preterm birth, preeclampsia, and stillbirth [2]. Complications associated with T2DM in pregnancy include preeclampsia (20%), early delivery secondary to medical necessity or spontaneous preterm birth (33%), and small or large-for-gestational age (LGA) infants (30%) [2]. Also, T2DM is associated with fetal programming of obesity and T2DM later in life [2]. Therefore, it is advantageous to maintain euglycemia to prevent fetal overgrowth. However, knowledge gaps exist regarding optimal therapy in pregnant women with overt T2DM to reduce morbidity.

The American Diabetes Association (ADA) [1] and the American Association of Clinical Endocrinologists [4] recommend that metformin therapy in the absence of contraindications be initiated with lifestyle intervention at the time of T2DM diagnosis. Metformin has excellent glycemic efficacy, offers decreased weight gain and decreased hypoglycemic episodes, and is well tolerated and reasonably priced [5].

Current recommendations for medical management of T2DM in pregnancy include frequent blood glucose monitoring combined with dietary management and insulin therapy to achieve euglycemia [1, 2]. However, use of oral hypoglycemic agents in gestational diabetes mellitus (GDM) has recently gained acceptance [6]. The efficacy of metformin for treatment of GDM was found to be comparable to insulin and had fewer side effects and increased patient preference [6]. Rowan and colleagues [7, 8] found that, in women with GDM, metformin alone or in combination with insulin was not associated with an increase in perinatal complications when compared to insulin alone. There were significantly fewer maternal hypoglycemic events (*p* = 0.008) and less maternal weight gain (*p* < 0.001) in women treated with metformin compared to insulin alone [7]. Also, a 2-year follow-up of infants of mothers enrolled in the study demonstrated no adverse effects and a reduction of visceral body fat [9]. In a retrospective, case-controlled study of women with T2DM, the researchers found comparable outcomes between women treated with metformin versus insulin [8, 10].

### Aims

The aims of this study are to compare the effectiveness and safety of insulin mono-therapy versus insulin plus metformin for treatment of T2DM complicating pregnancy. The primary study outcome is a composite adverse neonatal outcome, defined as the proportion of infants who have one or more of the following conditions at birth: 1) neonatal hypoglycemia, defined as a capillary blood glucose (CBG) < 40 or any hypoglycemia that requires intravenous (IV) fluid treatment; 2) birth trauma, defined as umbilical artery cord pH < 7.0, shoulder dystocia with either brachial plexus injury, clavicular fracture, humeral fracture, and/or > 3 maneuvers to relieve. Additional adverse neonatal outcomes include 3) hyperbilirubinemia that required infant phototherapy within the first 72 h after delivery; 4) delivery < 37 weeks’ gestation; 5) miscarriage (fetal loss < 20 weeks); 6) stillbirth (fetal loss > 20 weeks); 7) neonatal death (death prior to 28 completed days); 8) large-for-gestational age infant (birthweight >90th percentile for gestational age [GA]); and 9) small-for-gestational age (SGA) infant (birthweight <10th percentile for GA) or low birth weight (< 2500 g [gm]). In cases for which classification of the primary outcome is not straightforward, event status will be adjudicated by two independent members of an adjudication committee, with further review by a third member in the case of discordance.

There are two key secondary outcomes. The first is infant fat mass (%), as measured by anthropometrics. Birth weight, recumbent length, and head circumference will be abstracted from the chart. Right upper mid-arm circumference, right triceps, subscapular, and flank skinfolds will be measured by the study staff. The second is clinically relevant maternal hypoglycemia (CBG < 60) regardless of the presence of symptoms.

Additional exploratory secondary outcomes for the mother include maternal weight gain, defined as a change in weight from randomization until time of delivery, adjusted for GA at time of randomization; maternal side-effects such as nausea, vomiting, and diarrhea; maternal compliance determined by pill counts; maternal intention to breast or formula feed; adverse maternal outcomes including death, diabetic ketoacidosis, intensive care unit (ICU) admission, intubation, or renal failure; and maternal obstetrical complications including placental abruption or preeclampsia. Exploratory secondary outcomes for the neonate include neonatal metabolic complications other than hypoglycemia or hyperbilirubinemia, including polycythemia, neonatal intensive care unit (NICU) admission > 48-h length of stay, hyperbilirubinemia requiring phototherapy, intubation, or grade 3 or 4 intraventricular hemorrhage (IVH); and postpartum feeding experience at 30 days.

Additional study outcomes include measurement of maternal metabolic markers at 24–30 weeks’ gestation. One 10 ml (ml) tube of blood will be collected and centrifuged, and resultant serum will be frozen at − 80 °C for future analyses. Biomarkers planned to be measured include, but are not limited to, soluble fms-like tyrosine kinase-1, placental growth factor, leptin, and C-peptide. Other analyses will assess the role and interactions of clinical, biological, and biophysical factors on the occurrence and severity of maternal and neonatal outcomes.

## Methods

### Design

This is a phase III randomized double-blind clinical trial of insulin plus placebo versus insulin plus metformin for the treatment of overt T2DM complicating pregnancy. Women 10 weeks 0 days’ and 20 weeks 6 days’ gestation will be randomized and followed until 30 days after delivery. Neonate outcomes will be followed until 30 days of age.

All study subjects will receive standard surveillance and treatment for overt T2DM in pregnancy, which may include, but will not be limited to, a daily prenatal vitamin, supplemental folic acid, nutritional counseling and dietary recommendations, and insulin therapy as prescribed by their primary clinician (e.g., obstetrician or endocrinologist). The decision of the primary clinician on insulin dosing will be guided by standard guidelines based on the protocols that are used at the participating clinical trial centers. Instructions on capillary glucose monitoring will include a recommendation to test CBG at least four times a day. The goal of CBG parameters will be communicated to the subject by her primary clinician. The subject’s primary clinician will make adjustments to insulin dosing based on patient-recorded CBG measurements. Blood and urine sampling for hematologic, liver, and kidney function will occur at the primary obstetric clinician’s discretion. Likewise, ultrasound assessments of fetal size and well-being will occur at the primary obstetric clinician’s discretion. Fetal surveillance by nonstress test (NST; 30 min of continuous fetal heart rate monitoring) or biophysical profile (BPP; 30 min of ultrasound to monitor for fetal breathing, movement, flexion/extension, and amniotic fluid) will also occur at the primary obstetric clinician’s discretion. Routine care in labor and delivery will include, but will not be limited to, insulin therapy. Subjects will be instructed to hold the oral study agent (metformin or placebo) at the onset of labor, at induction of labor, or on the morning of a scheduled cesarean delivery. No metformin will be administered during labor or in anticipation of a scheduled cesarean delivery, when patients are asked to ingest nothing by mouth (NPO) for 12 h prior to the procedure.

Guidelines for insulin management have been created based on current strategies used at the study sites. Insulin dosing will be weight-based and administered as two to three daily injections. Insulin dose adjustments will be made at the discretion of the study site’s primary clinician using glycemic goals that are based on the ADA guidelines (Table 1). Women will test CBG levels daily while fasting and either 1 or 2 h postprandial, per the protocol at each clinical site, with goals listed in Table 2. The primary clinician will manage insulin dosing to achieve these goals as closely as possible.

**Table 1:** MOMPOD Study Insulin Dosing Guidelines by Trimester

	First Trimester	Second and Third Trimesters
Weight based dosing (U/kg/day)	0.4–0.7	0.7–1.2

**Table 2:** MOMPOD Study Capillary Blood Glucose Goals Based on ADA Guidelines

Fasting	1 Hour Postprandial	2 Hours Postprandial
< 95 mg/dl	< 140 mg/dl	< 120 mg/dl

In addition to the standard care described above, enrolled and randomized subjects will receive surveillance and treatment solely for study purposes. Women will be instructed to stop any other diabetes or oral hypoglycemic (OHA) medication and begin and/or continue insulin. All subjects will begin taking the study agent with the evening meal the day after randomization. This allows those who have been taking an oral agent previously to have at least a 24-h washout period. Daily administration of the study agent will be as follows: one capsule (metformin 500 mg or placebo) twice daily for 7–21 days based on study agent tolerance, followed by two capsules (metformin 1000 mg or placebo) twice daily. Drug tolerance is defined as the subject’s reported tolerance of the symptoms of nausea, vomiting and/or diarrhea and fewer than two episodes of self-reported hypoglycemia as manifested by a CBG < 60 mg/dL. Subjects will be instructed how to manage symptoms at home, including taking the study agent with meals and using over-the-counter medications to relieve symptoms such as nausea and diarrhea. If the subject has two or more self-reported hypoglycemia episodes (CBG < 60 mg/dL), she will be instructed to call her provider. Subjects will be instructed to increase the study agent after 7 days if they are tolerating the lower dose. If not, subjects will remain on the lower dose for up to three seven-day periods. Once increased to two capsules twice daily, the subject will be contacted weekly to determine tolerance at the higher dose. If the subject is unable to tolerate the study agent after a maximum of 21 days at either dose, she will be discontinued from the study agent. All subjects who discontinue the study agent will be followed through delivery.

If the subject reports intractable gastrointestinal (GI) symptoms or hypoglycemia during the phone calls to assess study agent tolerance, study staff will educate the subject on timing for taking the agent with food and will remind her of the medications she can use to relieve symptoms. If she experiences hypoglycemia, study staff will instruct her to contact her primary clinician to evaluate whether insulin dosing needs to be adjusted. Intractable symptoms are defined as any nausea, vomiting, or diarrhea despite the use of medications to decrease these symptoms. Study visits will coincide with prenatal visits to assess compliance and side-effects and dispense the study agent. Study visits are to occur every 4 weeks during selected, regularly scheduled, prenatal visits. Timing of study visits may vary based on the patient’s prenatal visit schedule, and can occur as often as once every 3 weeks. Study visits must occur at least once each 30 days.

Blood will be drawn at 24–30 weeks’ gestation to store serum for future studies. Chart abstraction will be conducted for maternal and delivery data (up until maternal discharge) and neonatal data (up until infant discharge or until 30 days of age, whichever comes first). Within 72 h of delivery, measurement of neonatal fat mass (i.e., anthropometrics) by skinfold caliper will be completed unless the infant is < 28 weeks’ gestation or the infant’s condition is unstable. Phone call or in-person contact with the mother after 30 days following delivery will be made to assess for serious adverse events and neonatal outcomes. The contact must be made within 45 days of delivery.

### Settings

The study is being conducted in partnership with University of North Carolina at Chapel Hill (UNC-CH) Women’s Clinic and Hospital, the Clinical Coordinating Center (CCC), UNC-CH Collaborative Studies Coordinating Center (CSCC), and Data Coordinating Center (DCC) all located in Chapel Hill, North Carolina as well as at the following sites: The Ohio State University at Columbus, Ohio; University of Pennsylvania, Philadelphia, Pennsylvania; University of California, San Diego Maternal Fetal Care Services and the Medical Offices South Women’s Health Services, San Diego, California; University of Alabama-Birmingham, Birmingham, Alabama; University of Utah Health Sciences Center, including the LDS Hospital, McKay-Dee Hospital Center, Utah Valley Regional Medical Center, and the Intermountain Medical Center in Salt Lake City, Utah; University of Texas Medical Branch, Galveston, Texas; University of Texas Memorial Hermann Hospital and the LBJ Hospital Houston, Houston, Texas; and Columbia University, New York, New York.

### Power analysis

We plan to randomize 1200 women. A 10% loss to follow-up will leave 1080 subjects with complete data and follow-up (540 per arm). We expect an additional 5–10% of subjects will discontinue the study agent for various reasons, including side-effects or intolerance, but will contribute follow-up data. This sample size gives adequate power over a range of expected primary outcome event rates, with type I error set at 0.044 (reduced from 0.05 for interim analysis), and maintains reasonable power under a conservative scenario, assuming that 10% of subjects immediately stop taking the study agent. Published data suggest that up to 30% of pregnant women with T2DM have an adverse neonatal outcome [11–13]. Our proposed sample size conservatively accounts for the possibility that the composite adverse neonatal outcome may be lower than anticipated. Our approach offers at least 89% power to detect an odds ratio of 0.60 for metformin versus placebo, assuming a primary outcome rate in the placebo group of 20% or more. Other scenarios, assuming a lower primary outcome rate in the placebo group or a smaller treatment effect, will yield ~ 80% power to detect a treatment difference.

### Sample

Inclusion criteria are as follows: maternal age 18–45 years old; informed verbal and written consent; singleton fetus with no known or suspected anomalies; overt type 2 diabetes, defined as either pregestational type 2 (overt) diabetes requiring medical treatment (e.g., oral agent or insulin), or overt diabetes diagnosed at < 20 weeks 6 days’ gestation, using either the one-step method (75 g glucose challenge test [GCT] with at least one abnormal value: fasting blood glucose (FBG) > 92, 1 h > 180 or 2 h > 153 g/dl) or the two-step method (50 g GCT > 135 mg/dl followed by 100 GCT with at least two values above thresholds: FBG > 90, 1 h > 180, 2 h > 155, 3 h > 140 mg/dl); or other method (e.g. A1C > 6.5%, OR fasting CBG > 126 mg/dL, OR random CBG > 200 mg/dL). Other inclusion criteria are a willingness to use insulin and study agent only and to use no other diabetes medical therapy while in the study as well as gestational age at randomization between 10 weeks 0 days and 20 weeks 6 days by menstrual dating confirmed by ultrasound or ultrasound alone.

Gestational age will be determined in the following manner and will be denoted as the study GA. The study estimated date of confinement (EDC), which will be based on the study GA, will not be revised once a determination has been made. If the pregnancy is conceived by in vitro fertilization, the study GA will be calculated from the date of embryo transfer and the embryo age at transfer. If the pregnancy is conceived spontaneously (including ovulation induction and artificial insemination), information from the last menstrual period (LMP) and earliest dating ultrasound will be used to assign study gestational age using the following algorithm: the first day of the LMP will be determined and a judgment made as to whether the patient has a certain LMP date. If the LMP date is certain, study GA will be determined by a comparison between the GA by LMP and by the earliest dating ultrasound. The first dating ultrasound must have been conducted before 20 weeks 6 days, by LMP. If the ultrasound confirms the GA calculated by LMP, as in Table 3, the LMP GA will be used as the study GA. Otherwise, study GA will be determined based upon the ultrasound measurement. If the LMP date is uncertain, measurement(s) obtained at the patient’s first dating ultrasound examination will be used to determine the study GA. The first dating ultrasound must be conducted before 20 weeks 6 days’ gestation.

**Table 3:** Determining Gestational Age

Gestational age at first ultrasound	Ultrasound agreement with last menstrual period
Up to 13 weeks 6 days	+ 5 days
14 weeks 0 days to 20 weeks 6 days	+ 7 days

Multiple gestation is an exclusion criterion; however, if a woman with a twin gestation spontaneously loses one fetus and retains the second fetus or is electively reduced to a singleton prior to 14 weeks, she will be eligible for participation. Exclusion criteria also include a suspected or known fetal structural or chromosomal abnormality, pre-existing renal disease with creatinine > 1.5 mg/dL, and medical contraindications to metformin as evidenced by a history of lactic acidosis. Additional exclusion criteria are acute liver disease or known liver abnormalities, such as acute viral hepatitis, aspartate aminotransferase, and/or alanine aminotransferase elevated more than twice the normal limit. Medical conditions that predispose a woman to gastrointestinal distress, such as Crohn’s disease, ulcerative colitis, irritable bowel syndrome (IBS), or celiac disease will further exclude a woman from participation in the study, as will current or past history of alcohol abuse and/or abnormalities in B12 metabolism, such as pernicious anemia, intrinsic factor deficiency, or prior partial or complete gastrectomy. Participation in another study that could affect primary outcome and/or randomization in the current study during a previous pregnancy are also exclusion criteria. Women will be excluded if their delivery is planned at a location other than a study hospital, if they are unwilling or unable to take insulin, and/or if they are unwilling or unable to swallow the study agent capsule or consume an inert ingredient in the study agent capsule. Finally, women will be excluded if another significant chronic medical or psychiatric illness would, in the investigators’ opinion, prevent participation in the study.

### Staff training and monitoring proficiency

The multiple principal investigators (MPIs), co-investigators, project coordinator, and research nurse will train study staff on all aspects of the study protocol (e.g., screening, recruitment, enrollment, randomization, study visit data collection, and specimen and data collection). Training sessions to orient staff to protocol and data collection forms will be held via webinar teleconference. Following training, all study staff must demonstrate proficiency in the protocol implementation as defined in the manual of operations (MOO). Once proficiency is demonstrated, individual study staff gain password-protected access to the electronic data entry website and can begin screening and recruitment of subjects. The DCC will generate data quality queries on an ongoing basis to inform sites of continued data collection proficiency.

One of the MPIs or her designee will train the UNC-CH project manager and research nurse and staff on the proper technique for infant measurements. A webinar teleconference, relying on a standardized system used in previous studies, will be developed for training all of the sites. Despite offering many benefits (e.g., low cost, easy to perform, little equipment required), anthropometrics are challenging, due to their vulnerability to measurement errors and lack of reliability. Unreliability can occur due to imprecision, such as measurement error variance due to intra- and inter-observer variability. Imprecision can arise from inadequate or improper training of personnel, difficulties in measurement of certain anthropometric characteristics, such as skinfolds, and instrumental or technical errors. We created a step-by-step approach in the MOO for all anthropometric measurements as well as a training video to teach measurement of bicep circumference and tricep, subscapular, and flank skinfolds. A duplicate measure program was developed and sites will be chosen at random by the DCC so that, for that entire month, all infant bicep circumferences and tricep, subscapular, and flank skinfolds will be measured by a second team member. Inter-rater reliability will be analyzed quarterly by calculating an inter-rater reliability coefficient. This allows timely feedback to be given to the site project coordinators and research nurses, and corrective measures will be taken quickly if reliability is not adequate.

### Ethical considerations

Each study site will develop a site-specific verbal and written consent form using the template consent form provided by the CCC. Each site will be responsible for obtaining Institutional Review Board approval and verbal and written informed consent for each enrolled subject. Each site will develop its own patient research authorization documents, as required by the Health Insurance Portability and Accountability Act Privacy Rule, following the guidelines of its own institution. A copy of the signed consent form will be provided to the enrolled subject. A person fluent in their language will enroll women who are not fluent in English. Both verbal and written informed consent and authorization will be obtained in that language. If this is not possible, the patient will be excluded from participation.

### Randomization and blinding

Randomization for enrolled subjects will occur at 10 weeks 0 days to 20 weeks 6 days of gestation. Enrolled subjects will be assigned to placebo or metformin in a 1:1 ratio in a permuted block design, stratified by study site and timing of diabetes diagnosis/baseline GA (four categories: pre-existing diagnosis and GA < 18 weeks, pre-existing diagnosis and GA ≥ 18 weeks, diagnosis during pregnancy and GA < 18 weeks, diagnosis during pregnancy and GA ≥ 18 weeks). The randomization sequence will be prepared and maintained centrally by the DCC. A statistician not otherwise involved with the study will create the randomization scheme, which will be implemented electronically within the web-based data management system. All study staff will be blinded to the allocation scheme until the end of the study. Emergency unblinding will be available around the clock for designated staff through the web-based data management system.

### Intervention

All subjects will receive insulin and metformin, 500 mg twice daily, or placebo, from enrollment through the first week (or up to 3 weeks based on tolerability), then metformin 1000 mg or placebo twice daily until delivery. A drug distribution center (DDC) will provide the blinded study agent (metformin and placebo) to the research sites. The study agent will be dispensed to subjects in a single bottle of 120 opaque capsules containing either 500 mg metformin or placebo matched to appearance and taste. Randomization will be performed via the web-based data management system, and a reporting feature within the system will indicate which bottle to dispense to the study subject at each study visit. Study visits are to occur at least every 4 weeks. Timing of study visits may vary based on the patient’s prenatal visit schedule, study visit scan occur as often as once each 3 weeks but must occur at least once each 30 days. The study agent will be bottled in 30-day quantities. Bottles will be labeled with the subject’s identification number. Subjects will be instructed to take the study agent, with food, as one capsule (500 mg metformin or placebo) twice a day, for 7 or up to 21 days, depending on study agent tolerance, then two capsules (1000 mg metformin or placebo) twice a day until delivery. If they miss a dose, subjects will be instructed to continue with dosing and not to double up to account for the missed dose. Subjects will be queried at study visits (coincident with prenatal visits) by study staff regarding compliance with the study regimen. Capsule counts will be conducted at each study visit. Independent of study participation the primary physician is responsible for all clinical treatment decisions including insulin dosing.

### Measurement

Study visits will coincide with clinical (prenatal) visits and occur approximately every three to 4 weeks. Timing of study visits may vary based on the patient’s prenatal visit schedule, but study visits must occur at least once each 30 days. At each visit, the medical record will be reviewed for intervening problem visits, hospitalizations, and insulin dosing adjustments, and data will be recorded. Staff will ask subjects about symptoms and events since the previous visit. Blood glucose logs will be reviewed and data abstracted (or the logs will be copied for later abstraction), and maternal weight and blood pressure will be recorded. Table 4 illustrates the forms that will be completed at each study visit.

**Table 4:** Schedule of assessments and forms

Study Encounter Name	Screening/ Enrollment Visit(s)	7-day Contact^a^	14-day Contact^a^	21-day Contact ^a^	28, 35, 42-day Contact ^a^	Prenatal Study Visits ^b^	Delivery Visit	30-day Postnatal Contact
Screening, informed consent, inclusion, exclusion, medical history, randomize	X							
Dispense study agent	X					XXX		
Dose escalation from 500 mg BID to 1000 mg BID		X^a^	X^a^					
Collect study bottle, pill count of returned bottle						XXX	X or plan to obtain	
Record total daily insulin dosing (from enrollment through delivery)	X	X	X	X	X	XXX	X	
Maternal side effects, symptoms, episodes of hypoglycemia, adverse events		X	X	X	X	XXX	X	
Maternal serious adverse events		X	X	X	X	XXX	X	X
Copy of glucose log						XXX	X or plan to obtain	
Maternal weight and blood pressure	X					XXX		
Blood draw						24–30 weeks’ gestation		
Maternal breastfeeding intention questionnaire						24–30 weeks’ gestation		
Measure infant anthropometrics							Within 72 h of delivery	
Collection of delivery and neonatal outcomes							X	X
Neonatal adverse events and serious adverse events							X	X
Maternal breastfeeding questionnaire postpartum								X

^a^ Dose is to be escalated at Day 7 and no later than Day 21 per subject’s study agent tolerance. Day 28, Day 35 and Day 42 contacts to occur only If indicated per subject’s study agent tolerance. All study agent tolerance contacts can be via telephone or in-person (such as during regularly scheduled pre-natal visits)

^b^ Study visits are to occur every 4 weeks during selected regularly scheduled pre-natal visits. Timing of study visits may vary based on the patient’s pre-natal visit schedule, but study visits must occur at least once each 30 days

### Data management

The DCC will be responsible for receiving all electronic data from the sites. Clinical center staff will enter data using the Carolina Data Acquisition and Reporting Tool web-based data management system. Data editing and validity checks and reports for missing fields are built into the system. The DCC has created an online tutorial to train staff on use of the data management system and all staff will be certified on data entry prior to completing data entry for the study.

### Interim analysis

We will perform a formal interim analysis to evaluate for early overwhelming efficacy after 50 and 75% of subjects have delivered and completed follow-up. We will also perform a formal interim analysis to evaluate for futility after 75% of subjects have delivered and completed follow-up. To assess early efficacy, the significance level for the interim and final analysis will be based on a Lan-DeMets α-spending function with O’Brien-Fleming boundaries in order to maintain the study-wise α level at 0.05. The α level will be 0.0031 at 50%, 0.0183 at 75%, and 0.044 at the final analysis. The exact α level at the interims will be determined based on the actual percent of data available at the interim. Assuming 90% power at the end of the study, there is a 26% chance of meeting the stopping boundary at the first interim and a 69% chance of meeting the stopping boundary at the second interim. Futility will be assessed at 75% based on conditional power. The Data Safety Monitoring Board (DSMB) may recommend that the study stop due to futility if the upper limit of the 80% confidence interval for conditional power does not exceed 50%.

A formal interim analysis for safety will be conducted beginning 1 year after enrollment and then every 6 months, to test whether the primary composite adverse neonatal outcome is significantly worse in the insulin/metformin group compared to the insulin/placebo group using a Lan-DeMets α-spending function with Pocock boundaries. Based on a one-sided alpha level of 0.025, the DSMB may consider stopping the trial at any of the six interim analyses if the *p*-value is < 0.007, corresponding to worse composite neonatal outcome in the insulin/metformin group. The DSMB will not evaluate for early efficacy except as described above at 75%.

Analyses will be performed by an independent, unblinded study statistician and presented to the DSMB, which will make recommendations regarding further conduct of the trial. The DSMB charter will include thresholds and rules for trial stoppage based on formal safety, efficacy, and futility limits.

### Data analysis

Analyses will follow the intention-to-treat principle, in which subjects will be analyzed within the group to which they were randomized, regardless of whether they received the assigned intervention or discontinued the study agent prior to delivery. Primary analyses will be based on the intention to treat population, which includes all randomized subjects who took at least one dose of the study agent. In addition, a secondary analysis of the primary outcomes will be completed for the per-protocol population, defined as a subset of the intention to treat population who took the assigned double-blind study agent until the time of delivery without any major protocol deviations. Major protocol deviations include violations in inclusion and exclusion criteria at enrollment and poor compliance with the study drug, defined as subjects taking less than 50% of the study drug. All major protocol deviations will be identified in a blinded fashion prior to database lock.

Descriptive statistics, chi-square tests, and analysis of variance will be used to characterize subjects enrolled and randomized to determine comparability of the two intervention groups at baseline for characteristics considered prognostic for the primary outcome, such as maternal body mass index (BMI), parity, and medical co-morbidities.

#### Primary outcome

Incidence of the primary outcome (i.e., composite adverse neonatal outcome) will be compared between treatment groups using logistic regression adjusting for study site, timing of diabetes diagnosis (pre-existing versus during pregnancy), gestational age at randomization (as a continuous measure), and baseline maternal BMI. Any sites with low enrollment may be pooled for statistical analyses. Odds ratio and 95% confidence interval will be calculated, as well as the number needed to treat to prevent the composite adverse neonatal outcome. Subjects who discontinue from the study and therefore have unknown primary outcomes will be considered as events. Statistical significance is determined relative to *p* = 0.044, to adjust for the formal interim analysis.

Sensitivity analysis of the primary outcome will include the following: 1) a complete case analysis, excluding subjects who discontinue from the study and therefore have an unknown primary outcome; 2) multiple imputation of missing values for the primary outcome, which assumes study drop-outs are missing at random; and 3) control-based multiple imputation, in which missing values for the primary events for the metformin arm are imputed based on the placebo event rates and analysis of the per-protocol population.

#### Additional analyses of the primary outcome

Pre-specified subgroup analyses will be conducted for factors that may be related to the outcome measures, including maternal BMI (obese versus non-obese), gestational age at time of randomization (10–< 14 weeks, 14–< 18 weeks, 18–< 21 weeks), timing of diabetes diagnosis (pre-existing versus during pregnancy), and by compliance level. Interaction tests will be used to determine whether the addition of metformin significantly differs across subgroups, such as obesity (BMI > 30 kg/m^2^) and reported compliance/adherence. *P*-values of 0.10 will be considered indicative of significant subgroup differences.

Other analyses to assess treatment effectiveness, adjusting for additional baseline patient characteristics (covariates), will be conducted. The objectives of these analyses are to estimate the influence of covariates on the outcome and to use covariates to improve the estimated difference between treatment groups. A stepwise logistic regression model will be used to identify and estimate the effect of multiple prognostic factors on the occurrence of the composite adverse neonatal outcome. These analyses will be considered exploratory in nature and will not be viewed as providing confirmatory tests of hypotheses. Descriptive analyses will also evaluate treatment group differences for each of the components of the composite outcome.

#### Secondary outcomes

There are two key secondary outcomes: infant fat mass (%), as measured by anthropometrics, and proportion of subjects with clinically relevant maternal hypoglycemia, defined as a CBG < 60, regardless of symptoms. Statistical significance of the treatment group comparisons for the key secondary outcomes will be evaluated in a step-down fashion in order to preserve the study-wise type 1 error rate. If the primary outcome is found statistically significant (at *p* < 0.044), then infant fat mass will be evaluated relative to *p* = 0.05. If found statistically significant, then the incidence of maternal hypoglycemia will be evaluated relative to *p* = 0.05. If any statistical test in this order fails to reach statistical significance, subsequent parameters will not be evaluated for statistical significance.

Analyses of all other secondary outcomes are considered exploratory in nature and will not be viewed as providing confirmatory tests of hypotheses. There will be no adjustment for multiple comparisons of the exploratory secondary outcomes, and *p*-values will be provided for descriptive purposes only.

Treatment groups will be compared for categorical secondary outcomes with logistic regression and for continuous secondary outcomes with analysis of covariance (ANCOVA). Variables that are not normally distributed will be assessed via nonparametric ANCOVA. All analyses will adjust for study site, timing of diabetes diagnosis (pre-existing versus during pregnancy), gestational age at randomization, and maternal BMI.

Further logistic regression and ANCOVA models will be used to identify and estimate the effect of multiple prognostic factors on secondary outcomes. Interaction tests will be used to determine whether the addition of metformin significantly differs across subgroups, such as obesity (BMI > 30 kg/m^2^), reported compliance/adherence, and others.

### Safety and adverse events

The steering committee and the DSMB are jointly responsible for safety monitoring. Site principal investigators, steering committee members, and/or their designated site study staff will conduct medical monitoring for unanticipated problems (UPs), adverse events (AEs), and serious adverse events (SAEs) and record and report them to their institutional IRB. The study PI responsible for correspondence will notify the DSMB, Federal Drug Administration (FDA), and IRBs regarding UPs, AEs, and SAEs as appropriate.

Detailed information concerning AEs and SAEs will be collected and evaluated throughout the trial. The DCC will report all AEs and SAEs to the DSMB. The DSMB will review all AEs, SAEs, and other interim safety data and will provide a report to the PIs and the IRBs. All SAEs are to be entered into the data monitoring system within 48 h of learning of the SAE.

#### Unanticipated problems involving risk to subjects or others

Any incident, experience, or outcome that meets all of the following criteria will be considered a UP. If an event is unexpected in nature, severity, or frequency (i.e., not described in study-related documents, such as the IRB-approved protocol or consent form, the investigators’ brochure, agent package insert, etc.) and if an event is related or possibly related to participation in the research (i.e., possibly related means there is a reasonable possibility that the incident experience or outcome may have been caused by the procedures involved in the research).

#### Maternal adverse events

An AE is an untoward medical occurrence, regardless of whether it is considered study-related, that occurs during the conduct of a clinical trial. Any change in clinical status (e.g., routine labs, X-rays, physical examinations, etc.) that is considered clinically significant by the study investigator is considered an AE. Any condition responsible for surgery will be documented as an AE if the condition meets the criteria for an AE. Any AE that results in hospitalization for 24 or more hours or prolonged hospitalization will be documented and reported as an SAE unless specifically instructed otherwise in this protocol. However, hospital admission alone is not automatically considered an AE. An AE does not include the following: preterm delivery, defined as delivery > 23 through < 36 weeks 6 days’ gestation or maternal procedures (e.g., surgery, endoscopy, tooth extraction) not related to the study. However, the condition that leads to the procedure is considered an AE. An AE also does not include pre-existing diseases or conditions present or detected prior to the start of study agent administration that do not worsen (e.g., hypertension, asthma, migraines) or a woman who is hospitalized when enrolled or who undergoes hospitalization or prolonged hospitalization for diagnostic or elective surgical procedures for a pre-existing condition, social and/or convenience reasons, or for infant delivery. AEs do not include overdose of either the study agent or concomitant medication without any signs or symptoms unless the subject is hospitalized for observation. Finally, the subject experiencing any event prior to taking her first dose of study agent will not be considered an AE.

#### Maternal serious adverse events

A serious AE is any AE for the mother that, as determined by the investigator or the sponsor, results in death. A life-threatening AE means that the study subject was, in the opinion of the investigator or sponsor, at immediate risk of death from the reaction as it occurred and required immediate intervention. However, inpatient hospitalization, prolongation of existing hospitalization, and maternal hospitalization for infant delivery are not considered reportable SAEs for this study. The definition of prolongation of an existing hospital stay includes maternal hospitalization for infant delivery for longer than 7 days and/or infant hospitalization for longer than maternal hospitalization. Any event that would result in a persistent disability. Any major congenital anomaly or birth defect involving a major organ system (e.g., a heart or brain defect). Minor congenital anomalies, such as choroid plexus cysts or renal pelvis dilation, are not considered major birth defects. An important medical event that may not result in one of the above outcomes, but that may jeopardize the health of the study participant or require medical or surgical intervention to prevent one of the outcomes listed in the above definition of serious event.

#### Infant adverse events and serious adverse events

The definition of AE and SAE is the same for infants as it is for mothers. Infant AEs and reportable SAEs for this study are detailed in Table 5 and based on the Eunice Kennedy Shriver National Institute of Child health and Human Development (NICHD) Pediatric Trials Network [14]. AEs that do not meet any of the criteria for serious will be regarded as non-serious AEs.

**Table 5:** Infant outcomes based on the Pediatric Trials Network

Adverse Event	Serious Adverse Event
Gastrointestinal	
NEC Intestinal perforation	NEC Bell stage II or III Perforation with intra-abdominal free air proceeding pneumatosis
Musculoskeletal	
Fracture with immobilization only	Fracture requiring surgical intervention
Pulmonary	
Respiratory failure requiring oxygen Pneumothorax present but no treatment required Apnea, any Pulmonary hypertension, any	Respiratory failure requiring mechanical ventilation Pneumothorax requiring intervention (e.g. chest tube, nitric oxide, milrinone, sildenafil)
Neurological	
Seizure, no medical treatment Intraventricular hemorrhage (IVH) grade I or II	Seizure requiring medical treatment IVH grade III or IV PVL on imaging
Cardiology	
Hypotension, no pharmacologic treatment Electrocardiogram (EKG)QT_c_ prolongation 460–485 ms Supraventricular tachycardia (SVT) resolving spontaneous or with noninvasive maneuvers (vagal maneuvers)	Hypotension requiring treatment with pressors EKG QT_c_ prolongation > 485 ms SVT requiring medical treatment or cardioversion
Infectious Disease	
Wound infection requiring topical treatment only	Other infection proven by culture (urine, blood, sputum, cerebrospinal fluid) requiring antimicrobials
Ophthalmologic	
Conjunctivitis requiring local treatment Retinopathy or prematurity (ROP), any stage not requiring treatment	Conjunctivitis requiring systemic intervention or treatment ROP, Any stage requiring surgical or medical treatment
Otolaryngology	
Hearing impairment	Confirmed hearing loss, unilateral or bilateral
Miscarriage	
Pregnancy loss < 12 weeks, 0 days	

#### Adverse event reporting period

The study period during which AEs must be reported is defined as the period from the initiation of any study procedures to the end of the study treatment follow-up. The study treatment follow-up period for the mother is defined as until discharge from the hospital. For the infant, the treatment follow-up period is through 30 days of life. In addition, AEs and SAEs for the mother and infant must be reported through 30 days after the last dose of study agent.

A pre-existing condition is one that is present at the start of the study. A pre-existing condition will be recorded as an AE if the frequency, intensity, or the character of the condition worsens during the study period. Local site PIs will follow all unresolved AEs until the events are resolved, the subject is lost to follow-up, the subject is withdrawn from the study, or the AE is otherwise explained. Subjects will be instructed to notify the study staff at their local institution if they experience an event that might be related to the study agent, even if it occurs following completion of participation. Primary clinicians will also be instructed to notify study staff if their patient experiences an event that might be related to the study agent.

There are no clinical laboratory tests performed as part of this study, but clinical labs may be measured as standard care for the patient. A clinical laboratory abnormality will be documented as an AE if the following conditions are met: the laboratory abnormality is not refuted by a repeat test to confirm the abnormality and the abnormality suggests a disease and/or organ toxicity that requires active management, such as discontinuation of the study agent, more frequent follow-up assessments, or further diagnostic evaluation and management.

#### Reporting of serious adverse events and unanticipated problems

MPIs and local site PIs will conform to the reporting timelines, formats, and requirements of the various entities to which they are responsible. For any maternal, fetal, or neonatal deaths or other life-threatening events, they will notify immediately (within 24 h of the site learning of the death) and enter as much information onto the SAE form as can be completed. A copy of the patient’s medical record pertaining to the event should be made and a death certificate requested, when available. If an autopsy is performed, a copy of the report should be collected as well. All sites need to report deviations per their site IRB protocol.

#### Sponsor reporting: Notifying the FDA

The contact PI is required to report certain study events in an expedited fashion to the FDA. These written notifications of adverse events are referred to as the Investigational New Drug (IND) safety reports. The following describes the safety reporting requirements, by timeline, for reporting and associated type of event based on the date that the data was entered into the online data entry system if it is uncommon and strongly associated with study agent exposure otherwise once determined to qualify for reporting by the study coordinator. Any study event that meets SAE requirements, is determined to be related to the study agent, is unexpected (as defined under IND reporting requirements), is fatal or life-threatening (as defined under IND reporting requirements), or a previous adverse event that was not initially deemed reportable but is later found to fit the criteria for reporting must be reported within 7 days. Any study event that meets SAE requirements and is uncommon and known to be strongly associated with exposure to the study agent, determined to be related to the study agent, is unexpected (as defined by IND reporting requirements), is serious (as defined under IND reporting requirements) but not fatal or life-threatening, or a previous adverse event that was not initially deemed reportable but is later found to fit the criteria for reporting will be reported within 15 days.

#### Stopping rules

Study stopping rules will be based on DSMB review. Individual stopping rules include delivery and maternal side-effects that cannot be managed with oral medication. However, data collection will continue through study completion (30 days following last dose of study agent for mother and 30 days after delivery for infant) to ascertain maternal and infant outcomes.

#### Medical monitoring

Each site PI will oversee the safety of the study at his/her site. This safety monitoring will include careful assessment and appropriate reporting of AEs as noted above, as well as the construction and implementation of a site data- and safety-monitoring plan. Medical monitoring includes a regular assessment of the number and type of serious AEs. If a subject develops an SAE, the subject’s obstetric care provider, in collaboration with the site PI and the trial unblinded medical monitor, will ascertain the safety of continuing the intervention.

#### Unblinded MedicaI monitor

The trial has a designated unblinded medical monitor who is otherwise uninvolved with the study and not a member of the DSMB. The medical monitor will review each reported SAE to evaluate the PI’s assessment of the relationship to the study agent and expectedness and to ensure that the site PI follows the patient appropriately. In cases of emergency, unblinding materials will be made available to the unblinded medical monitor or a study PI if the unblinded medical monitor is not available. The medical monitor can share unblinded information about the study agent with other healthcare professionals, including the site PI, if unblinding of the site PI is determined necessary by the study PI and the medical monitor.

#### Independent data safety monitoring board

An independent DSMB will provide oversight to ensure that the trial accrues at a sufficient rate and that the safety and privacy of all study subjects is protected. DSMB members are not involved in any aspect of the trial operation. The DSMB will function under a charter that specifies guidelines for operation. Detailed information concerning adverse events will be collected and evaluated throughout the conduct of the trial. The DSMB will review all treatment-emergent adverse events, all SAEs, and other interim safety data, and will provide a report of recommendations to the steering committee and to the local IRBs.

In addition to SAE review by the unblinded medical monitor, the DSMB will receive a monthly update of SAEs and a periodic full report of safety data per DSMB decision to review study progress, to monitor the conduct of the study for safety concerns, and to conduct the formal interim analyses for efficacy, futility, and safety analysis. The DSMB will make recommendations about the conduct of the study for safety concerns. The DSMB could recommend to continue the study, suggest a protocol modification, temporarily halt enrollment to allow further safety investigation, or to end the study early. To maintain the blind for the entire study team, an independent statistical analysis team at the CSCC will produce the closed session report with unmasked treatment groups. Closed reports will be maintained behind a firewall, with access allowed only to the unblinded team.

## Discussion

The MOMPOD study will provide high quality, contemporary evidence for management of overt T2DM complicating pregnancy to improve neonatal outcomes. This study has the potential to change medical management of women with overt T2DM and pregnancy not only in the United States but also globally since metformin is cost-effective and tolerated well in nonpregnant women.

## Abbreviations

ADA: American Diabetes Association
AE: Adverse event
ANCOVA: Analysis of covariance
BID: Twice a day
BMI: Body mass index
CBG: Capillary blood glucose
CCC: Clinical coordinating center
CSCC: Collaborative studies coordinating center
DCC: Data coordinating center
DDC: Drug distribution Center
DSMB: Data and Safety Monitoring Board
EDC: Expected Date of Confinement
EKG: Electrocardiogram
FBG: Fasting blood glucose
FDA: Food and Drug Administration
GA: Gestational age
GCT: Glucose challenge test
GDM: Gestational diabetes mellitus
GI: Gastrointestinal
GM: Gram
HIPAA: Health Insurance Portability and Accountability Act
IBS: Irritable bowel syndrome
ICU: Intensive care unit
IND: Investigational new drug
IRB: Institutional review board
IVH: Intraventricular hemorrhage
LGA: Large for gestational age
LMP: Last menstrual period
MOO: Manual of operations
NICHD: National Institute of Child Health and Human Development
NICU: Neonatal intensive care unit
NPO: Nothing per oral
NST: Non-stress test
OHA: Oral hypoglycemic agent
PI: Principal investigator
ROP: Retinopathy of prematurity
SAE: Serious adverse event
SGA: Small for gestational age
SVT: Supraventricular tachycardia
T2DM: Type 2 diabetes mellitus
UNC-CH: University of North Carolina at Chapel Hill
UP: Unanticipated problem
US: United States
